# Validation of A 3D-printed simulator for training in endoscopic injection of bulking agent for vesicoureteral reflux: a pilot study

**DOI:** 10.1007/s00464-024-11081-6

**Published:** 2024-07-29

**Authors:** Maria Escolino, Annalisa Chiodi, Giovanni Esposito, Claudia Di Mento, Luisa Florio, Mauro Porcaro, Ciro Esposito

**Affiliations:** 1 Division of Pediatric Surgery, Federico II University Hospital, Via Pansini 5, 80131 Naples, Italy; 2 CEINGE Advanced Biotechnologies, Naples, Italy; 3 Division of Pediatric Radiology, Federico II University Hospital, Via Pansini, 5, 80131 Naples, Italy

**Keywords:** Surgical simulation, Model, Training, Vesicoureteral reflux, Assessment, Surgery

## Abstract

**Background:**

Simulation-based training plays a significant role in surgical education, especially in minimally invasive pediatric surgery and urology. This study aimed to evaluate a novel 3D-printed model as training tool for endoscopic injection of bulking agent.

**Methods:**

Forty-three attendees and ten teaching faculty members were invited to complete a post hoc questionnaire after completing training sessions using the Fish Tank Simulation Model (FTSM). The survey consisted of a 7-question 5-point Likert scale to assess the model’s realism (face validity) and its effectiveness as training tool (content validity).

**Results:**

Regarding the training status, 20/53 (37.7%) participants were fellow and/or specialist in pediatric surgery and 33/53 (62.3%) were surgeons in training. Their level of confidence in endoscopic injection procedure was defined as novice (< 10 procedures per year) in 33/53 (62.3%), intermediate (10–20 procedures per year) in 10/53 (18.9%), and expert (> 20 procedures per year) in 10/53 (18.9%). Regarding both face validity and content validity assessments, no statistically significant differences were found between scores given by novice vs intermediate/expert groups. Similarly, no statistically significant differences emerged between scores given by participant vs faculty groups assessing the content validity of the FTSM. The FTSM was considered a good teaching tool for beginners by 44/53 (83%) and for pediatric surgeons/urologists by 38/53 (71.7%).

**Conclusions:**

The 3D-printed Fish Tank Simulation Model proved to be a valuable, high-fidelity, easily accessible, cost-effective, hygienic, and domestic-use training tool for pediatric surgeons/urologists conducting the procedure. The model’s user-friendly design and realistic environment enhanced learning opportunities for trainees, regardless of their experience level or training status. Nevertheless, further development is necessary, particularly in enhancing the realism of the ureteral hiatus and reproducing more complex anatomy, to make it beneficial for the training of advanced surgeons.

**Supplementary Information:**

The online version contains supplementary material available at 10.1007/s00464-024-11081-6.

Simulation-based training plays a significant role in surgical education, especially in pediatric minimally invasive surgery and urology [1, 2]. Several surgical specialties are employing training using surgical simulators beyond the confines of the operating room. For instance, there is widespread acknowledgment that simulators serve as effective educational aids and enable the assessment of technical proficiency in laparoscopic surgery [3–5].

Recent advancements highlight the integration of various emerging simulation modalities in urology training. These include operable 3D-printed models, robotic surgery simulation, and online simulation [2].

At present, 3D printing finds applications in urology for educating trainees and patients, facilitating surgical planning, crafting urological equipment, and exploring bioprinting [6, 7].

Endoscopic treatment by injection of dextranomer/hyaluronic acid (Dx/HA) is currently the first-line therapy of pediatric vesicoureteral reflux (VUR) in many institutions worldwide [8, 9]. The Dx/HA needle injection procedure involves a brief, tightly constrained process, demanding the precise implantation of a concentrated 1–3 mL paste into an exceedingly small and delicate tissue area [10, 11]. This procedure allows minimal space for technical adjustments, intraoperative corrections, or revisions. Despite these challenges, there is currently no established simulator with validation for learning and practicing the skills applicable to this procedure that can be transferred effectively to the operating room [12].

A 3D-printed Fish Tank Simulation Model (FTSM) was constructed to allow physicians new to the endoscopic injection technique for VUR to become familiar with the equipment, set-up and use, hand positioning, posture, and practice visualization of injection through a cystoscope and camera system.

This study aimed to evaluate this novel 3D-printed model as training tool for endoscopic injection of bulking agent.

## Materials and methods

### Simulator design

The Fish Tank Simulation Model (FTSM) is a comprehensive model that provides a realistic simulation of a pediatric female bladder and distal ureters. Lazarus 3D, Inc. (Philomath, OR, USA) created the bladder and ureter models using 3D-modeling software based on computerized tomography (CT) scan imaging and printed them using a silicone-based material. The key features of the FTSM are the Clear Acrylic Box with a fitted lid and anti-slip grip that is water tight and transparent for easy observation during training; the Female Anatomy to provide a practical training environment, simulating the urethral opening and a 0.5-inch female urethra, bladder, and bladder holder; and the Modular Wet Ureters to extend the useful life of the trainer kit. The Modular Wet Ureters measure 60 mm (length) × 25 mm (width), and the ureteral orifice has a diameter of 6 mm. They have Injection Site Markings, represented by pink internal dots to mark the injection sites and aid in accurate practice.

It was Palette Life Sciences that provided the ureter inserts which were used during the study.

### Simulator costs

The main structure of the model is represented by 3D-printed female urethra, bladder, and bladder holder in watertight box and is reusable for repeated times. This structure costs 2.500 USD. The consumable materials consist of Modular Wet Ureters, which are dual sided and intended for 2 independent uses, maximizing the training potential of each model. Each Modular Wet Ureter for 2 independent uses has a cost of 70 USD.

### Equipment needs

**Fig. 1:**
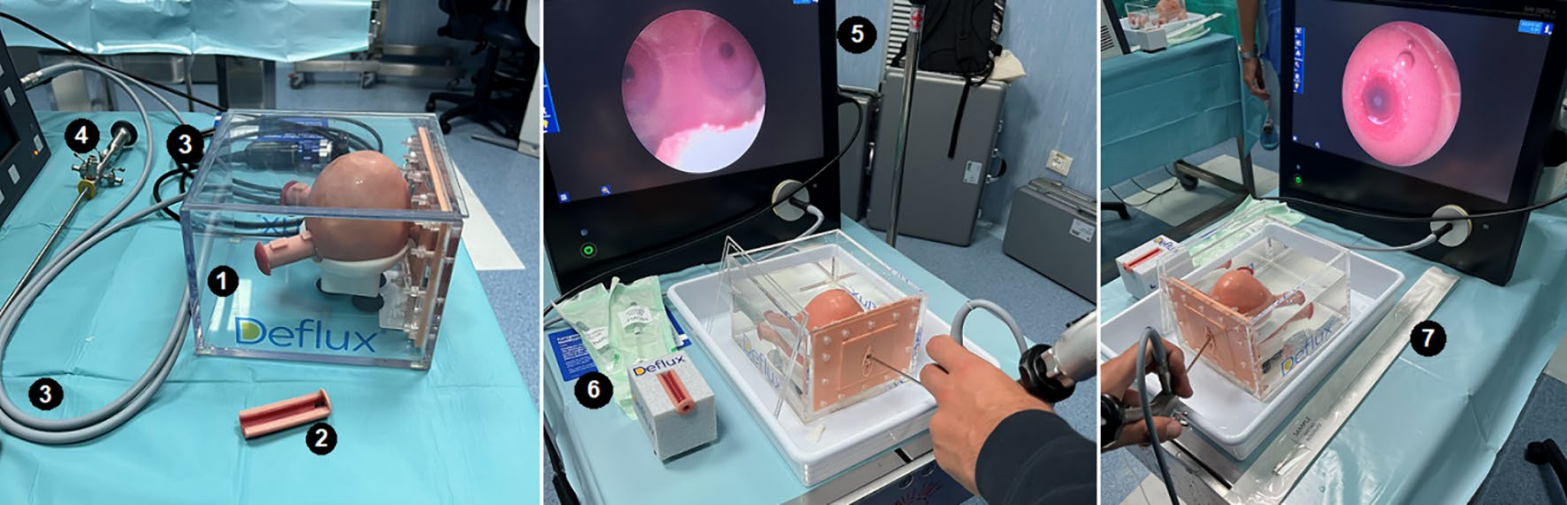
Equipment: (1) FTSM in watertight box; (2) simulator ureter inserts; (3) camera and light source; (4) pediatric straight working channel scope; (5) monitor; (6) Dx/HA syringe; (7) Dx/HA needle

The requirements were FTSM in watertight box and urethral plug; simulator ureter inserts; water source to fill the FTSM; waterproof sheeting – to protect furniture; towels – for water absorption; small cup – to remove excess water from FTSM (Fig. 1).

The procedures required Dx/HA syringe; Dx/HA needle; endoscopic equipment tower with camera and light source; pediatric straight working channel scope; and IV pole with bag of fluid (Fig. 1).

### Prepare the FTSM

1. Place tray with lip on table or bed with one towel open on top of tray;
2. Fill the FTSM with urethral plug with water to top of simulated bladder;
3. Depress dome of bladder to express air – (air will obscure camera vision);
4. Insert ureteral insert by matching notch in bladder with notch in insert;
5. Have physician or equipment manager set-up endoscopic tower and scope;
6. Attach fluid source to scope port; and
7. Prepare Dx/HA needle and syringe.

### Using the FTSM

1. Position learner in front (or to side) of the FTSM;
2. Practice proper posture, stance, and body alignment for optimal camera view;
3. Insert the scope;
4. Insert the Dx/HA needle; and
5. Complete injection.

Video reproduces the endoscopic injection procedure performed using the FTSM.

### Study design

The study enrolled 43 trainees and 10 faculty members participating in a 2-day endoscopic skill training course. During the course, they performed 8 h of training with endoscopic injection using the FTSM under the supervision of faculty members. The 8-h training course included an initial theoretical part where the model was illustrated and a video of the procedure to be performed on the model was shown to the attendees, and a second practical part in which each attendee took turns performing a procedure on the model. At the end of the course, all were asked to complete a post hoc questionnaire survey. The survey consisted of a 7-question form to assess the model’s realism (face validity) and its effectiveness as training tool (content validity) (Appendix 1).

We performed a subgroup analysis of the scores given by novice vs intermediate/expert or by participant vs faculty on the face and content validity of the FTSM.

The study received appropriate Institute Review Board (IRB) approval.

### Statistical analysis

Statistical analysis was carried out using the Statistical Package for Social Sciences (SPSS Inc., Chicago, Illinois, USA), version 13.0. The associations between qualitative variables were measured by the chi-square test, and quantitative variables were measured with the parametric Student’s t-test. *p* < 0.05 was considered statistically significant.

## Results

### Participant demographics

Regarding the training status, 20/53 (37.7%) participants were fellow and/or specialist in pediatric surgery, and 33/53 (62.3%) were surgeons in training. Their level of confidence in endoscopic injection procedure was defined as novice (< 10 procedures performed per year) in 33/53 (62.3%), intermediate (10–20 procedures performed per year) in 10/53 (18.9%), and expert (> 20 procedures performed per year) in 10/53 (18.9%). The most adopted bulking agent in their experience was Deflux®, as reported by 47/53 (88.7%) of responders.

All data are reported in Table 1.

**Table 1:** Demographic data of participants

Data	Number of participants, *n* (%)
Medical career status	
1-year trainee	8/53 (15.1)
2-year trainee	8/53 (15.1)
3-year trainee	10/53 (18.9)
4-year trainee	5/53 (9.4)
5-year trainee	2/53 (3.8)
Fellow	2/53 (3.8)
Specialist	18/53 (34)
Level of confidence in injection procedure for VUR	
Novice (< 10 procedures performed per year)	33/53 (62.3)
Intermediate (10–20 procedures performed per year)	10/53 (18.9)
Expert (> 20 procedures performed per year)	10/53 (18.9)
Bulking agent adopted in common practice	
Deflux®	47/53 (88.7)
Vantris®	4/53 (7.5)
Macroplastique®	3/53 (5.7)
Dexell®	5/53 (9.4)
Other	0/53

*VUR* vesicoureteral reflux

### Face validity assessment

**Fig. 2:**
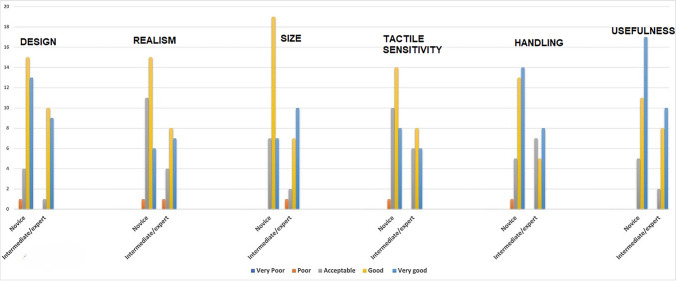
Face validity assessment of the Fish Tank Simulation Model (FTSM) in novice group vs intermediate/expert group

Trainees subjective scores (mean ± SD), based on a 5-point Likert scale (1 = very poor; 2 = poor; 3 = acceptable; 4 = good; 5 = very good), were grouped according to the level of experience (Fig. 2). No statistically significant differences were found between scores given by novice group vs intermediate/expert group on the face validity of the FTSM (Table 2).

**Table 2:** Subgroup analysis of face validity assessment of the Fish Tank Simulation Model (FTSM)

Parameter	Novice group score (mean ± SD)	Intermediate/expert group score (mean ± SD)	*p* =
Design	4.21 ± 0.11	4.4 ± 0.13	0.57
Realism	3.78 ± 0.15	4.05 ± 0.20	0.52
Size	4.0 ± 0.13	4.3 ± 0.10	0.60
Tactile sensitivity	3.87 ± 0.11	4.0 ± 0.15	0.47
Handling	4.21 ± 0.18	4.05 ± 0.10	0.48
Usefulness	4.36 ± 0.17	4.4 ± 0.15	0.57

The FTSM was considered a good teaching tool for beginners by 44/53 (83%) and for pediatric surgeons/urologists by 38/53 (71.7%).

### Content validity assessment

Immediately after performing the procedure on the simulator, participants were asked to evaluate the ability of the simulator to realistically simulate the actual procedure.

**Fig. 3:**
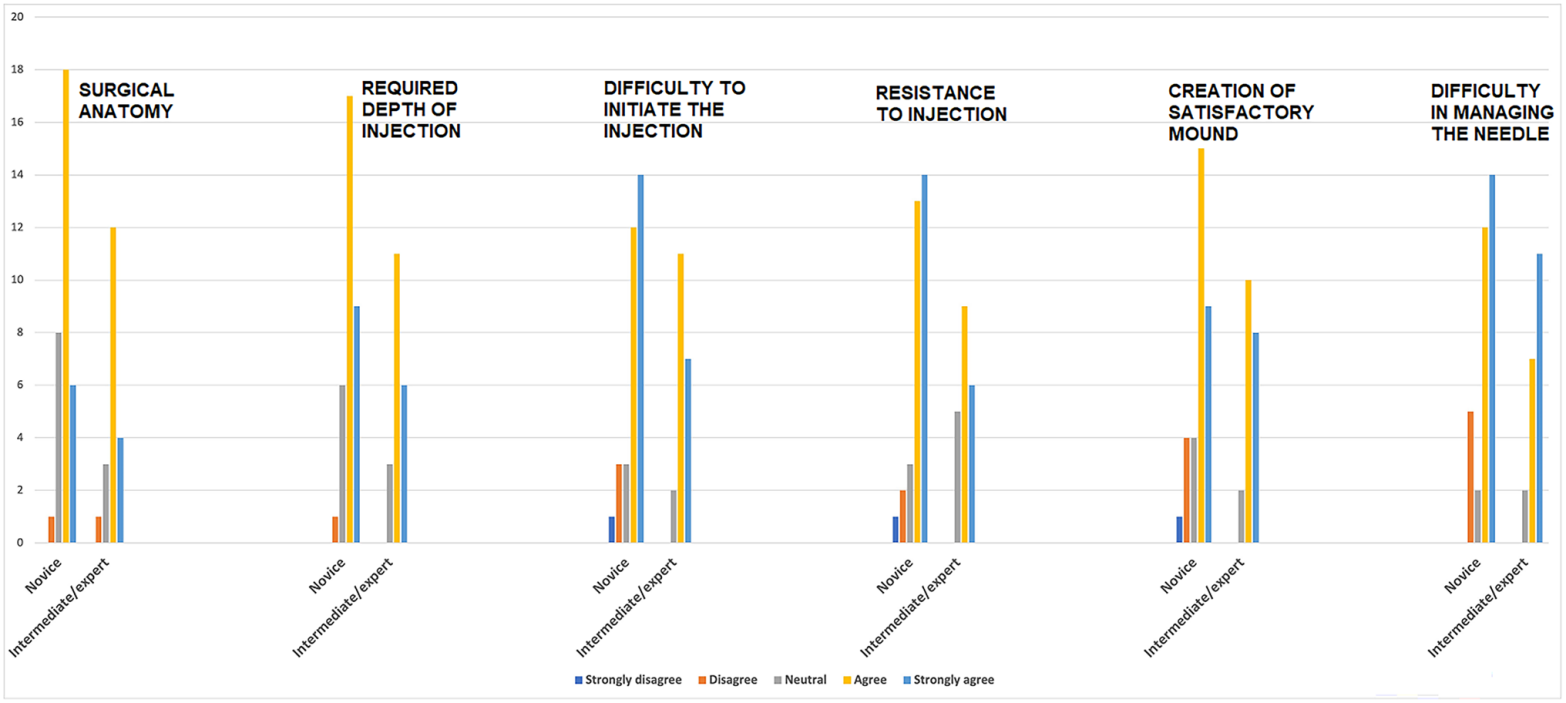
Content validity assessment of the Fish Tank Simulation Model (FTSM) in novice group vs intermediate/expert group

The subgroup analyses revealed no statistically significant differences between scores given by novice vs intermediate/expert group on the content validity of the FTSM (Table 3) (Fig. 3).

**Table 3:** Subgroup analysis (novice vs intermediate/expert) of content validity assessment of the Fish Tank Simulation Model (FTSM)

	Strongly disagree, *n* (%)	Disagree, *n* (%)	Neutral, *n* (%)	Agree, *n* (%)	Strongly agree, *n* (%)
Surgical anatomy					
Novice (*n* = 33)	0	1 (3)	8 (24)	18 (54.5)	6 (18.2)
Intermediate/expert (*n* = 20)	0	1 (5)	3 (15)	12 (60)	4 (20)
*p* =	N/A	0.71	0.42	0.69	0.86
Required depth of injection					
Novice (*n* = 33)	0	1 (3)	6 (18.2)	17 (51.5)	9 (27.3)
Intermediate/expert (*n* = 20)	0	0	3 (15)	11 (55)	6 (30)
*p* =	N/A	0.43			
Difficulty to initiate the injection					
Novice (*n* = 33)	1 (3)	3 (9.1)	3 (9.1)	12 (36.4)	14 (42.4)
Intermediate/expert (*n* = 20)	0	0	2 (10)	11 (55)	7 (35)
*p* =	0.43	0.16	0.91	0.18	0.59
Resistance to injection					
Novice (*n* = 33)	1 (3)	2 (6.1)	3 (9.1)	13 (39.4)	14 (42.4)
Intermediate/expert (*n* = 20)	0	0	5 (25)	9 (45)	6 (30)
*p* =	0.43	0.26	0.11	0.68	0.36
Creation of satisfactory mound					
Novice (*n* = 33)	1 (3)	4 (12.1)	4 (12.1)	15 (45.4)	9 (27.3)
Intermediate/expert (*n* = 20)	0	0	2 (10)	10 (50)	8 (40)
*p* =	0.43	0.10	0.81	0.74	0.33
Difficulty in managing the needle					
Novice (*n* = 33)	0	5 (15.2)	2 (6.1)	12 (36.4)	14 (42.4)
Intermediate/expert (*n* = 20)	0	0	2 (10)	7 (35)	11 (55)
*p* =	N/A	0.06	0.59	0.92	0.37

When comparing the scores given by participant vs faculty group, no significant differences were found as well (Table 4).

**Table 4:** Subgroup analysis (participant vs faculty) of content validity assessment of the Fish Tank Simulation Model (FTSM)

	Strongly disagree, *n* (%)	Disagree, *n* (%)	Neutral, *n* (%)	Agree, *n* (%)	Strongly agree, *n* (%)
Surgical anatomy					
Participant (*n* = 43)	0	1 (2.3)	10 (23.3)	22 (51.2)	10 (23.3)
Faculty (*n* = 10)	0	1 (10)	1 (10)	8 (80)	0
*p* =	N/A	0.25	0.35	0.09	0.09
Required depth of injection					
Participant (*n* = 43)	0	1 (2.3)	8 (18.6)	21 (48.8)	13 (30.2)
Faculty (*n* = 10)	0	0	1 (10)	7 (70)	2 (20)
*p* =	N/A	0.62	0.51	0.22	0.51
Difficulty to initiate the injection					
Participant (*n* = 43)	1 (2.3)	3 (6.9)	4 (9.3)	17 (39.5)	18 (41.9)
Faculty (*n* = 10)	0	0	1 (10)	6 (60)	3 (30)
*p* =	0.62	0.38	0.94	0.23	0.48
Resistance to injection					
Participant (*n* = 43)	1 (2.3)	2 (4.6)	5 (11.6)	17 (39.5)	18 (41.9)
Faculty (*n* = 10)	0	0	3 (30)	5 (50)	2 (20)
*p* =	0.62	0.48	0.14	0.54	0.19
Creation of satisfactory mound					
Participant (*n* = 43)	1 (2.3)	4 (9.3)	6 (13.9)	18 (41.9)	14 (32.6)
Faculty (*n* = 10)	0	0	0	7 (70)	3 (30)
*p* =	0.62	0.31	0.20	0.10	0.87
Difficulty in managing the needle					
Participant (*n* = 43)	0	5 (11.6)	3 (6.9)	15 (34.9)	20 (46.5)
Faculty (*n* = 10)	0	0	1 (10)	4 (40)	5 (50)
*p* =	N/A	0.25	0.74	0.76	0.84

## Discussion

Simulation models have become increasingly popular, emerging as a crucial component in the training and skill development of medical students and residents prior to their engagement in the operating room [1, 13]. Simulation training provides an opportunity for practical experience in a lifelike environment, eliminating the potential harm to patients [12]. In this low-stress setting, trainees can concentrate on honing surgical skills without the anxiety associated with actual surgical outcomes. An additional distinctive aspect of the simulator is its encouragement of mistakes, recognizing their importance in the learning process and fostering the development of strategies to prevent or address procedural complications [13–15].

Over the last years, surgical simulators have been created and widely adopted as an integral element of skills training for various minimally invasive surgical procedures [2–5].

The needle injection of bulking agent is a brief, highly precise procedure lasting 1–2 min, involving the confined implantation of 1–3 mL of bulking agent into an extremely small and delicate tissue area [10]. There is limited room for technical compensation, adjustment, intraoperative correction, or revision. In addition, the expected caseload for non-referral centers is not high; therefore, a simulation model to help the learning curve is of high value.

Soltani et al. [16] described a porcine bladder-based Dx/HA injection simulator. However, some small differences existed between the porcine model and human ureteral orifices making the injection more challenging.

To date, there is no evidence confirming the efficacy of an animal model for developing endourological skills among residents and urologists. In addition, factors such as the availability of labs, ethical considerations, and the relatively high cost of surgery sessions on animal models need to be considered. Indeed, efforts are needed to establish an ideal training model that is cost effective, efficient, and can effectively translate into results in the operating room.

The cost effectiveness of this model is linked to its reusability, as opposed to animal models, which require additional costs related to the acquisition and disposal of animals. In addition, the FTSM has the advantage to be usable in each setting, whereas live tissues must be used only in equipped wet laboratories, thus, requiring additional costs for leasing laboratory spaces. Analyzing the literature, low-cost simulation models for rigid and flexible cystoscopy were already constructed and validated [17]. Nevertheless, to date, there are no training simulators for injection of bulking agents in the urology field.

Our 3D-printed simulator for endoscopic injection realistically simulates the actual clinical procedure. This model facilitates the introduction of specialized equipment with Dx/HA, hitting on 3 senses: Hearing, Seeing, and Feeling. All faculty members strongly agreed that the model was useful to develop hand–eye coordination, tactile sensitivity, and handling. It may also enhance conversation on operating room set-up, patient positioning, and required equipment. Technical skills that can be mastered with this simulation practice, include proper posture, use of different hand positions, proper syringe needle assembly, proper angle of needle and mucosal puncture, needle size opening and mucosal depth, coaptation of tunnel, hydrodistension, proper volume of material to inject to create satisfactory mound. It also allows physicians to experience feeling of bulking agent injection force and finding the target zone to inject the material. It aids to train with the different techniques of injection such as STING, HIT, and double HIT. Finally, it shows implant bulking, lifting, and support at ureter orifice.

Water management is the clumsiest issue with this simulator. Indeed, it is necessary to provide the dry lab with waterproof sheeting to protect furniture, towels for water absorption, small cup to remove excess water from the FTSM. In addition, the model could be further improved in different aspects such as introducing different configurations of ureteral hiatus, adding the element of hydrodistension, or improving the quality of the biomaterial.

Although previous studies [18] have employed ‘experts’ to assess the content validity of a simulation model, our subgroup analyses showed no significant differences in scores between novice and intermediate/expert groups or participant vs faculty groups. This might be due to the model’s user-friendly design and realistic environment, which enhances learning opportunities for trainees regardless of their experience level or training status.

Limitations of the present study are the small sample size, absence of a control group, and lack of test–retest.

The trainees assessed the models in random order, which could potentially affect the outcomes of the study. Given the absence of a gold standard for assessing the performance of individuals administering Dx/HA injections and the associated simulation curriculum, the calculation of criterion validity is unfeasible. Consequently, our evaluation relies on discriminant and convergent validity to assess the effectiveness of this teaching tool.

At present, we lack data on the real-life performance of the procedure of subjects exposed to the simulation curriculum. Moreover, information regarding the health outcomes of treated patients is not available. Nevertheless, additional studies are required to define the impact of skill acquisition more precisely in real-life scenarios and assess its sustainability.

Additionally, we acknowledge that in the literature, there is an open-source model for laryngeal injections in a 3D-printed larynx [19]. Currently, our model is not available as open source, but the next step, following its validation, could be to turn it into an open-source model, thus, allowing people to replicate it in their own institutions.

In conclusion, the 3D-printed Fish Tank Simulation Model proved to be a valuable, high-fidelity, easily accessible, cost-effective, hygienic, and domestic-use training tool for enhancing the performance of pediatric surgeons/urologists conducting the procedure. Nevertheless, further development is necessary, particularly in enhancing the realism of the ureteral hiatus and reproducing more complex anatomy, to make it beneficial for the training of advanced surgeons.

### Electronic supplementary material

Below is the link to the electronic supplementary material.

Supplementary file1 (MP4 164130 kb)

## Appendix 1

Questionnaire survey

**Table:**

Questions	Answers
1. At what level in your medical career are you?	•1-year trainee •2-year trainee •3-year trainee •4-year trainee •5-year trainee •Fellow •Specialist surgeon
2. At what level in VUR injection procedure are you?	•Novice (< 10 procedures per year) •Intermediate (10–20 procedures per year) •Expert (> 20 procedures per year)
3. Which bulking agent do you adopt in your practice?	•Deflux® •Vantris ® •Macroplastique ® •Dexell ® •Other
4. This simulation model for endoscopic injection accurately simulates the following items: •Surgical anatomy •Required depth of injection •Difficulty in initiating the injection •Resistance to injection •Optimal pressure to create the mound •Optimal volume to create the mound •Creation of satisfactory mound •Difficulty in managing the needle	For each item, 5-point Likert scale: 1. Strongly disagree 2. Disagree 3. Neutral 4. Agree 5. Strongly agree
6. Give your score to the following characteristics of the simulation model tested: •Design •Realism •Size •Tactile sensitivity •Handling •Usefulness	For each item, 5-point Likert scale: 1. Very poor 2. Poor 3. Acceptable 4. Good 5. Very good
7. This simulation model for endoscopic injection is a good support for teaching: •Beginners •Pediatric surgeons/urologists	For each item, 5-point Likert scale: 1. Strongly disagree 2. Disagree 3. Neutral 4. Agree 5. Strongly agree
